# The presence of human papillomavirus (HPV) in placenta and/or cord blood might result in Th2 polarization

**DOI:** 10.1007/s10096-017-2958-z

**Published:** 2017-03-21

**Authors:** H.-M. Koskimaa, A. Paaso, M. J. P. Welters, S. Grénman, K. Syrjänen, S. H. van der Burg, S. Syrjänen

**Affiliations:** 1 Department of Oral Pathology, Institute of Dentistry, Faculty of Medicine, University of Turku, Lemminkäisenkatu 2, 20540 Turku, Finland; 2 Department of Pathology, Turku University Hospital, University of Turku, Turku, Finland; 3 Department of Obstetrics and Gynaecology, Turku University Hospital, Turku, Finland; 4 Department of Clinical Research, Biohit Oyj, Helsinki, Finland; 5 Department of Clinical Oncology, Leiden University Medical Center, Leiden, The Netherlands

## Abstract

The purpose of this study was to evaluate if an early exposure to human papillomavirus (HPV) during the prenatal period or infancy could result in HPV16-specific T helper (Th) responses resembling those of adults with HPV-induced lesions. We tested HPV16-specific cell-mediated immunity (CMI) in children born with HPV-positive umbilical cord blood and/or placenta or having persistent oral HPV infection and in constantly oral HPV-negative controls. Peripheral blood mononuclear cells from 33 children from the Finnish HPV Family Study cohort (mean age 14.7 years) were stimulated with peptide pools covering the amino acid sequence of the HPV16 E2, E6, and E7 proteins. Lymphocyte proliferation, secretion of cytokines (IFN-γ, TNF-α, IL-2, IL-4, IL-5, IL-10, IL-17A), and the frequency of Foxp3+ regulatory T-cells were determined in relation to the HPV DNA status during a 14-year follow-up. 73.6% of cases and 85.7% of controls responded against HPV16 E2, while reactivity against E6 was found in 10.5 and 35.7%, respectively. The proliferative response against E6 and E7 was more frequent in controls than in cases (*p* = 0.047). No HPV16-specific CMI response or antibodies were detected in two children with persistent oral HPV16. The profiles of induced cytokines indicated higher levels of IL-5, IL-10, and IL-17A in children with HPV DNA in placenta and/or cord blood than in other children. HPV16-specific CMI is common in HPV DNA-negative children. The cytokine profile in children infected with HPV16 during early life suggests that the viral dose and/or specific environment created by the placenta may have significant impact on the type of HPV-specific immunity.

## Introduction

Human papillomavirus (HPV) infection is generally regarded as a sexually transmitted disease. However, several studies, including those of our own [14, 18, 22, 31], have shown that HPV can be detected in the oral mucosa of newborn babies and young, sexually inexperienced children, suggesting that alternative, non-sexual routes for HPV infection are plausible.

We have shown a strong concordance between the HPV genotypes detected in the mother’s genital tract and her offspring’s oral samples [14], implying that the HPV-infected mother is the most probable source of HPV infection of the newborn. This view is also supported by others [8, 10, 16, 20, 24] reporting vertical transmission rates between 18.2 and 53.3% in the mother–baby pairs. In line with this, we reported highly concordant serum antibody profiles for HPV-L1 antigens in the mother–baby pairs [14].

Vertical transmission of HPV from a mother to her infant is still under debate, and several possible modes have been implicated: during (i) fertilization, (ii) pregnancy (prenatal), or (iii) delivery (perinatal). We recently provided evidence for prenatal transmission, as we found that the presence of HPV in placenta and/or in cord blood increased the risk of the newborn to carry HPV DNA in the oral mucosa [odds ratio (OR) = 14.0; 95% confidence interval (CI), 3.7–52.2; *p* = 0.0001 and OR = 4.7; 95% CI, 1.4–15.9; *p* = 0.015, respectively) [14, 25]. This implicates that the placenta may play an important role in the transmission of HPV, not unlike several other human viruses [42]. Indeed, studies on the role of the placenta and other maternal microbiomes as regulators of neonates’ microbiomes have increased only recently [28], attesting the view that the fetus can be exposed to microbiota during pregnancy, with long-term sequels in their health. Thus, the placenta has been suggested to act as a site where the translocated maternal oral microbes are presented to the fetal immune system, resulting in the development of prenatal tolerance to the maternal microbiome [43].

The significance of early encounters with HPV in the pre- or perinatal period is unclear. Because of the immaturity of the immune system of the fetus and infant [7], any early exposure to HPV might possess a major impact in the development of HPV-specific immunity, subsequent HPV infections, and their eventual progression. However, HPV-specific immunity in children has remained a practically uncharted area. Prompted by the observations of HPV infections among children, we decided to study HPV-specific immunity during childhood.

Unexpectedly, many children aged 12–14 years had HPV16 E2-, E6-, and E7-specific immunoreactivity [13, 15]. Previous studies have reported much lower frequency of HPV16-specific cell-mediated immunity (CMI) in adults [3, 4, 34, 35, 37, 38, 40, 41]. In adults, differences in the memory T helper (Th) cell reactivity against HPV16 E2, E6, and E7 oncoproteins have been found between HPV-positive and HPV-negative subjects. In healthy individuals, the Th responsiveness against HPV16 E2 and E6 seems to be more common and accompanied by the secretion of both type 1 and type 2 cytokines in a mixed pattern than in subjects with HPV16-induced disease.

Given the previously shown close association between HPV DNA in the placenta and/or cord blood and oral HPV carriage of the child, we hypothesized that those children exposed to HPV via placenta or cord blood and/or with persistent oral HPV might present HPV16-specific Th responses resembling those of adults with HPV-induced lesions, highly divergent from constantly HPV-negative controls. We, therefore, studied the HPV16 E2-, E6-, and E7-specific immune response and surveyed other characteristics, including vertically acquired, persisting HPV, and maternal or self-produced HPV-specific antibodies.

## Materials and methods

### Children

The current study is part of the Finnish HPV Family Study, which is a longitudinal cohort study conducted at the Department of Oral Pathology, Institute of Dentistry, University of Turku and the Department of Obstetrics and Gynecology, Turku University Central Hospital. Originally, 329 pregnant women in their third trimester of pregnancy and all their newborns were enrolled in the study between 1998 and 2001, as described previously [15, 22, 23]. The newborns were actively followed up for 6 years (mean 54.9 months, median 62.4 months). Of these children, 33 were selected according to the follow-up data: 16 children, who had either HPV DNA detected in umbilical cord blood and/or placenta and/or HPV DNA detected in oral mucosa continuously (>24 months), and 17 children, who stayed constantly HPV-negative during the follow-up. Of these children, 26 were recalled personally to give blood samples for CMI analyses and oral scrapings for HPV testing. Seven of the selected 33 children had been tested previously [13, 15]. A written informed consent was obtained from all these children and their parent(s). In total, 11 of 18 girls had received prophylactic bivalent HPV vaccination (Cervarix) before the sample collection, whereas none of the 15 boys had. In addition to the samples collected during the present study, we also had access to all previous follow-up data on these children.

The authors acknowledge the concept of the Minimal Information About T cell Assays (MIATA) framework [2, 12].

### HPV DNA detection and HPV antibody screening

Oral scrapings from the children were taken from the buccal mucosa of both cheeks and superior and interior vestibule using a cytobrush (Medscand) [22] at birth, at the age of 3 days, at 1, 2, 6, 12, 24, 36, and 72 months, and at the last time point, which was, on average, 14.7 years. DNA was extracted from scraping samples using the high salt method [19, 30]. First, the high-risk HPV types were detected by nested polymerase chain reaction (PCR) with MY09/MY11 and GP05+/GP06+ primers. HPV genotyping was done with the Multimetrix® assay (Multimetrix, Regensburg, Germany), as described earlier [32], which detects the following low-risk (LR) HPV types: 6, 11, 42, 43, 44, and 70 and high-risk (HR) HPV types: 16, 18, 26, 31, 33, 35, 39, 45, 51, 52, 53, 56, 58, 59, 66, 68, 73, and 82. The median fluorescence intensity (MFI) of at least 100 beads was computed for each bead set in the sample. The cut-off value for each HPV probe individually was defined by 1.5 × MFI + 15 MFI. Blood samples for the detection of HPV-specific serum antibodies were collected from children at the age of 1, 2, 6, 12, 24, and 36 months and sent for analysis to the German Cancer Research Center (DKFZ), Heidelberg, Germany. Serum antibodies for the major capsid protein L1 of HPV types 6, 11, 16, 18, and 45 were analyzed by multiplex HPV serology, based on glutathione S-transferase fusion-protein capture on fluorescent beads [36]. The results were analyzed and compared with each other by using two different cut-off levels (i.e., 200 MFI and 400 MFI) [32].

### Blood samples for CMI studies

Venous blood samples (54 mL) were collected in sodium-heparin collection tubes from children at the indicated time points and the peripheral mononuclear cells (PBMCs) were isolated by Ficoll (GE Healthcare Life Sciences, Uppsala, Sweden) density gradient, as described previously [15, 37, 39]. Of these PBMCs, ∼13 × 10^6^ cells were subjected to the short-term lymphocyte stimulation test (LST) to determine the proliferative capacity of T-cells against the indicated proteins of HPV16 [15, 39]. Nine milliliters of blood collected into a clotting tube was used to isolate serum by centrifugation for 7 min at 1800 g, which was used in the LST, as described previously [15, 39]. The remaining PBMCs were cryopreserved in 80% Fetal Bovine Serum (FBS, Biowest, EU quality) and 20% DMSO (Merck, Darmstadt, Germany) and stored in liquid nitrogen.

### HPV16 peptides

Eight peptide pools of HPV16 peptides were used to determine the proliferative capacity of HPV16-specific T-cells, and the cytokine polarization and frequency of HPV16-specific Foxp3+ regulatory T-cells, as described previously [15, 37, 39]. 30 (or 35) amino-acid-long peptides with 14- (or 15-)mer overlap covering the HPV16 E2, E6, and E7 protein sequences were synthesized by the solid-phase peptide synthesis (SPPS) method with >95% purity (ChinaPeptides Co., Shanghai, China). Two pools of E2 peptides (E2.1 and E2.2) consisted of 12 or 11 (30-mer) peptides, respectively. Four pools of E6 (E6.1–E6.4) and two pools of E7 peptides (E7.1 and E7.2) consisted of two 32-mer or 35-mer peptides, respectively [15]. Memory response mix (MRM) stock solution (50×), consisting of tetanus toxoid, 0.75 fL/mL (Statens Serum Institut, Copenhagen, Denmark), tuberculin purified protein derivative (PPD), 5 μg/mL (Statens Serum Institut), and *Candida albicans*, 0.015% (Greer Laboratories, Lenoir, NC, USA) was used as a positive control for the proliferation assays and cytokine production capacity of the PBMCs [15, 39].

#### Determination of the proliferative capacity of HPV16-specific T-cells by the short-term LST

LST was performed as described previously [15, 39]. Briefly, PBMCs were cultured in eight replicative wells in the presence of the indicated peptide pool at a final concentration of 5 μg/mL per peptide. PBMCs cultured with MRM were used as a positive control and with no antigen (medium only) as a background control. After culturing for 6 days, supernatant from the replicative wells was pooled and stored for cytokine analysis. The proliferation of cells were determined by the [^3^H]-thymidine incorporation assay. The cut-off value for counts per minute (CPM) values was determined by the average plus 3 × standard deviation (SD) of the eight medium-only control wells. The stimulation index (SI) was calculated as the average of the eight tested wells divided by the average of the medium-only control wells. The proliferative response was defined as positive if the CPM values of at least six of the eight wells were above the cut-off value and if the SI was ≥3.

### Cytokine polarization analysis

The supernatants from PBMC cultures were analyzed using the Cytometric Bead Array (CBA) human enhanced sensitivity flex set system (BD Biosciences, Temse, Belgium), according to the manufacturer’s instructions. In this array, the levels of IFN-γ, TNF-α, IL-2, IL-4, IL-5, IL-10, and IL-17A were determined, as previously described [4, 15, 39]. The detection limits for the cytokines were based on standard curves complying with the limit of 274 fg/mL, as described by the manufacturer. The positive antigen-induced cytokine production was defined as a cytokine concentration >2× the concentration of the medium-only control.

#### Identification of HPV16-specific CD4+CD25+Foxp3+ regulatory T-cells

Regulatory T-cell identification was performed as previously described [9, 15, 39]. Briefly, PBMCs were cultured with the same peptide pools as used in the LST at a final concentration of 5 μg/mL per peptide. Cells cultured with MRM were used as a positive control and cells without antigen (medium only) were used as a background control. After 7 days of culturing, the cells were harvested and stained with surface markers CD25 (1:25) (anti-CD25 FITC, clone M-A251, BD Pharmingen, San Diego, CA, USA), CD4 (1:100) (anti-CD4-APC, clone RPA-T4, BD Pharmingen), CD8 (1:30) (anti-CD8 PerCP-Cy5.5; clone SK1, BD Pharmingen), and intracellular marker Foxp3 (PE anti-human FOXP3, clone 206D, BioLegend) or isotype control (PE Mouse IgG1, κ Isotype Ctrl, BioLegend). After staining, the cells were acquired by the flow cytometer BD FACSCalibur (BD Biosciences). Analysis was performed by using Flowing Software, version 2.5.0 (Cell Imaging Core, Turku Centre for Biotechnology, Turku, Finland). The fluorescent intensity of MRM-stimulated and medium-only control cells was used to set the gates for the other samples. An antigen-induced alteration in the population percentage was defined as a change of at least 2× the corresponding percentage in the medium-only controls.

### Statistical analysis

The means of secreted cytokine concentrations and proliferative responses of all groups were analyzed using one-way analysis of variance (ANOVA). The Bonferroni correction was used to control for multiple comparisons between the groups. All statistical tests were two-sided and declared significant at a *p*-value ≤0.05. All statistical analyses were run using the IBM SPSS® (IBM, Inc., New York, USA) software package (IBM SPSS Statistics for Windows, version 22.0.0.1).

## Results

### Children’s oral HPV DNA status and HPV serology

All 33 children were actively followed up from birth until the age of 6 years and passively thereafter, until the collection of the present samples taken now from the children with a mean age of 14.7 years (median 14.9 years, range 11.7–16.2 years). The HPV DNA status of the oral mucosa was determined at birth, at the age of 3 days, 1, 2, 6, 12, 24, 36, and 72 months, and, finally, at 14.7 years. HPV serology to genotypes 6, 11, 16, 18, and 45 was determined at the same time points from the age of 1 to 36 months. Fourteen children stayed constantly HPV-negative during follow-up and HPV16-negative at final time point, and were considered as controls, while 19 children, who had either HPV DNA detected in umbilical cord blood and/or placenta and/or HPV DNA detected in oral mucosa either continuously (> 24 months) or HPV16 only at the final time point, were defined as cases. Tables 1 and 2 show the detailed individual follow-up data.

**Table 1:** The oral HPV and HPV antibodies of the case children from the baseline to 14.7 years. Subgroups: (I) HPV DNA-positive placenta and detectable serum HPV antibodies during the first 2 months after birth; (II) HPV DNA-positive placenta without any HPV antibodies during the first 2 months; (III) HPV DNA-positive cord blood without an HPV DNA-positive placenta; (IV) persisting oral HPV (>24 months) during the active follow-up of 72 months, without an HPV DNA-positive placenta of cord blood; and (V) negative for oral HPV at study points during 72 months follow-up, oral HPV16 at the final time point. Oral HPV was tested at every time point and defined as persistent if the same HPV genotype was detected in at least two time points within a period of 24 months. Serum antibodies were detected at the age of 1, 2, 6, 12, 24, and 36 months. The numbers in the boxes indicate the detected HPV type

Subgroup	ID	HPV DNA in cord blood	HPV DNA in placenta		Mother before delivery (genital HPV and antibodies)	Child										Persistent oral HPV DNA	HPV vaccination
						At birth	At the age of										
							3 days	1 month	2 months	6 months	12 months	24 months	36 months	72 months	14.7 years		
I	102		HPV16	Oral HPV DNA	NEG	HPV16	NEG	HPV16	HPV16	NEG	NEG	NEG	NEG	HPV16	NEG	HPV16	YES
				HPV antibodies	6, 11			6	6	neg	neg	neg	NA				
	205		HPV16	Oral HPV DNA	HPV16	NEG	NEG	HPV16	HPV16	NEG	NEG	NA	NA	NEG	NEG		YES
				HPV antibodies	16			16	neg	neg	neg	NA	NA				
II	104		HPV83	Oral HPV DNA	NEG	HPV16	NEG	HPV16	HPV16	NEG	NEG	HPV16	HPV16	HPV16	NEG	HPV16	NO
				HPV antibodies	neg			neg	neg	neg	neg	neg	neg				
	202	HPV16	HPV16	Oral HPV DNA	NEG	HPV16, 33, 59	NEG	HPV16	HPV18	NEG	NEG	NEG	NEG	HPV33, 59	NEG	HPV33, 59	NO
				HPV antibodies	neg			neg	neg	neg	NEG	6, 11	6				
	203	HPV16	HPV16	Oral HPV DNA	NEG	HPV16, 33	NEG	NEG	HPV16	NEG	NEG	NA	NA	NA	NEG		YES
				HPV antibodies	6			neg	neg	neg	neg	NA	NA	NA	NEG		
	204	HPV6	HPV6	Oral HPV DNA	HPV6, 59	NEG	NEG	NEG	NEG	HPV6	NEG	NEG	NEG	NEG	NEG		NO
				HPV antibodies	6, 16, 18, 45			NA	neg	neg	6	neg	neg				
	206	HPV6	HPV6	Oral HPV DNA	HPV6	HPV6	NEG	HPV6	NEG	NEG	NEG	NEG	NEG	NA	NEG		YES
				HPV antibodies	neg			NA	neg	neg	neg	neg	neg				
III	101	HPV39		Oral HPV DNA	NEG	HPV39	NA	NA	NA	NA	NA	NA	NA	HPV39	NEG	HPV39	NO
		HPV16		HPV antibodies	neg			NA	NA	NA	NA	NA	NA				
	102	HPV16		Oral HPV DNA	NEG	NEG	NEG	HPV16	HPV16	HPV16	NEG	NEG	HPV16	NEG	HPV16	HPV16	NO
				HPV antibodies	6			neg	neg	neg	neg	neg	neg				
	201*	HPV16		Oral HPV DNA	NEG	NEG	NA	NA	NA	NEG	NEG	NEG	NEG	NEG	NA		NO
				HPV antibodies	11			11	NA	neg	neg	neg	neg				
IV	301*			Oral HPV DNA	HPV16, 31, 42	HPV31	NEG	NEG	NEG	NEG	NEG	HPV70	HPV18	HPV	NA	HPV31	NO
				HPV antibodies	6, 16			neg	NA	neg	neg	16	neg				
	302*			Oral HPV DNA	HPV31	HPV31	HPV6	HPV33	NEG	NEG	NEG	NEG	NEG	HPV31	NA	HPV31	NO
				HPV antibodies	6			NA	6	neg	neg	neg	6				
	303			Oral HPV DNA	NEG	HPV33	NEG	NEG	NEG	NEG	NEG	NEG	HPV16	HPV33	NEG	HPV33	NO
				HPV antibodies	16			16	NA	NA	18	NA	neg				
	304			Oral HPV DNA	NEG	HPV16	NEG	NEG	NEG	NEG	HPV16	NEG	HPV16	HPV16	HPV11	HPV16	NO
				HPV antibodies	6, 16			6, 16	neg	neg	neg	neg	neg				
	305			Oral HPV DNA	NEG	NEG	NEG	NEG	NEG	HPV16, 59	NA	NA	NA	HPV16	HPV6, 16	HPV16	YES
				HPV antibodies	neg			neg	neg	neg	NA	NA	NA				
	306			Oral HPV DNA	NEG	HPV45	NEG	NEG	NEG	NEG	NEG	HPV16	HPV16	HPV45	NEG	HPV45	NO
				HPV antibodies	neg			neg	neg	neg	neg	neg	6				
	011			Oral HPV DNA	NEG	NEG	NEG	NEG	NEG	NEG	NEG	NEG	NEG	NEG	HPV16	HPV45	NO
				HPV antibodies	6			6	6	neg	neg	neg	neg				
	013			Oral HPV DNA	NEG	NEG	NEG	NEG	NEG	NEG	NEG	NEG	NEG	NA	HPV16		YES
				HPV antibodies	16			16	6, 16	neg	6, 11	6	neg				
	014			Oral HPV DNA	NEG	NEG	NEG	NEG	NEG	NEG	NEG	NEG	NEG	NA	HPV16		YES
				HPV antibodies	neg			16	NA	6	neg	6, 11	6, 11				

*NEG* no HPV DNA detected; *neg* no HPV antibodies; *NA* no sample available

*Child has been tested previously [12, 13]

**Table 2:** The oral HPV and HPV antibodies of the control children, who remained constantly negative for oral HPV from baseline to 1.7 years. Oral HPV was tested at every time point, serum antibodies at the age of 1, 2, 6, 12, 24, and 36 months. The numbers in the boxes indicate the detected HPV type

ID		Mother before delivery (genital HPV and antibodies)	Child										HPV vaccination
			Baseline	At the age of									
				3 days	1 months	2 months	6 months	12 months	24 months	36 months	72 months	14.7 months	
001	Oral HPV DNA	NEG	NEG	NEG	NEG	NEG	NEG	NEG	NEG	NEG	NEG	NEG	NO
	HPV antibodies	6			NA	6	neg	neg	16	6, 16			
002	Oral HPV DNA	NEG	NEG	NEG	NEG	NEG	NEG	NEG	NEG	NEG	NEG	NEG	NO
	HPV antibodies	neg			NA	neg	neg	neg	6	neg			
003*	Oral HPV DNA	NEG	NEG	NEG	NEG	NEG	NEG	NEG	NEG	NEG	NA	NA	NO
	HPV antibodies	6, 11, 16, 18			NA	neg	neg	neg	neg	neg			
004	Oral HPV DNA	NEG	NEG	NEG	NEG	NEG	NEG	NEG	NEG	NEG	NEG	NEG	YES
	HPV antibodies	6			6, 16	6, 16	6	6	6, 16	6			
005	Oral HPV DNA	NEG	NEG	NEG	NEG	NEG	NEG	NEG	NEG	NEG	NEG	HPV11	NO
	HPV antibodies	neg			neg	neg	neg	neg	neg	6			
006	Oral HPV DNA	NEG	NEG	NEG	NEG	NEG	NA	NA	NA	NA	NA	NEG	YES^a^
	HPV antibodies	6		NA	NA	NA	NA	NA	NA	NA			
007	Oral HPV DNA	NEG	NEG	NEG	NEG	NEG	NEG	NEG	NEG	NEG	NEG	NA	YES
	HPV antibodies	6, 16			neg	neg	neg	6	6	neg			
008*	Oral HPV DNA	NEG	NEG	NEG	NEG	NEG	NEG	NEG	NEG	NEG	NEG	NA	NO
	HPV antibodies	6, 16, 18			11, 16	11, 16	neg	neg	neg	neg			
009	Oral HPV DNA	NEG	NEG	NEG	NEG	NEG	NEG	NEG	NEG	NEG	NEG	NEG	NO
	HPV antibodies	6, 16			6, 16	neg	neg	neg	6, 16	6			
010	Oral HPV DNA	NEG	NEG	NEG	NEG	NEG	NEG	NEG	NEG	NA	NA	NEG	YES
	HPV antibodies	6, 11, 16, 18			NA	NA	neg	6	neg	NA			
012*	Oral HPV DNA	NEG	NEG	NEG	NEG	NEG	NEG	NEG	NEG	NEG	NA	NA	NO
	HPV antibodies	16			16	neg	neg	neg	neg	6			
015	Oral HPV DNA	NEG	NEG	NEG	NEG	NEG	NEG	NEG	NEG	NEG	NEG	NEG	NO
	HPV antibodies	neg			neg	NA	neg	neg	neg	neg			
016	Oral HPV DNA	18, 45	NEG	NEG	NEG	NEG	NEG	NEG	NA	NA	NA	NEG	NO
	HPV antibodies	6, 11, 16, 18			6, 11, 16	6, 11	neg	NA	NA	NA			
017*	Oral HPV DNA	16	NEG	NEG	NEG	NEG	NEG	NEG	NEG	NA	NA	NA	NO
	HPV antibodies	16			neg	neg	neg	6	6	NA			

*NEG* no HPV DNA detected; *neg* no HPV antibodies detected; *NA* no sample available

^a^One of three doses of HPV vaccination received before the final time point

*Child has been tested previously [12, 13]

Among cases, 16 of 19 children were tested for HPV at the last follow-up visit, and six of them had oral HPV: HPV11 was detected in one, HPV16 in four, and HPV6/16 coinfection in one child. Overall, oral HPV of any genotype was detected in all case children during the follow-up of 14.7 years, except one (ID201), who was HPV cord blood positive but remained oral HPV DNA-negative during the entire follow-up. In the control group, 9 of 14 children were tested at the last follow-up visit, one having oral HPV11. None of these 14 control children had any HPV DNA detectable in their oral mucosa at any study point during the original 72 months of follow-up. The bivalent HPV vaccination showed no significant effect on HPV detection at the last visit.

Altogether, HPV antibodies for genotypes 6, 11, and 16 were found in 13 of 19 case children, nine of them having antibodies during the first 2 months of life, i.e., prior to the emergence of their own antibody production, indicating the maternal origin of these HPV antibodies. Among the controls, HPV antibodies were detected in 11 of 14 children, and six of them had antibodies during the first 2 months of life. Interestingly, of the 11 children with persistent HPV infection, ten had the same HPV type detected already at delivery or during the first 2 months, implicating that HPV acquired at very early age may predispose to HPV persistence.

### Short-term LST and HPV-specific CD4+CD25+Foxp3+ regulatory T-cell detection

**Fig. 1:**
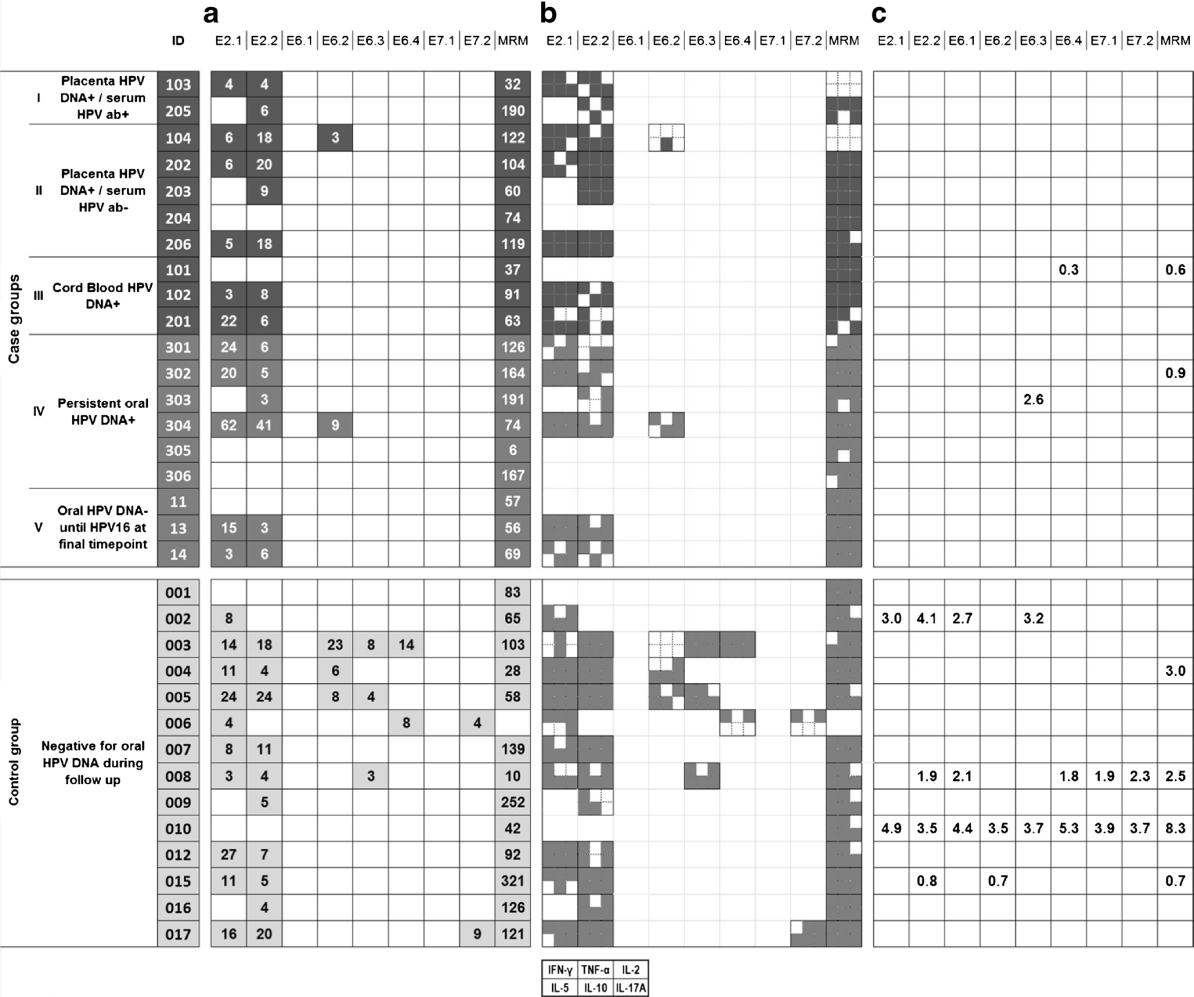
Results from lymphocyte stimulation test (LST), cytokine polarization, and Foxp3 assay. **a** Results of the HPV16-specific LST. Only positive responses are marked with *gray boxes* and the stimulation index is shown below each peptide pool. Memory response mix (MRM) was used as a positive control. **b** Supernatants from the LST were analyzed for the presence of cytokines IFN-γ, TNF-α, IL-2, IL-5, IL-10, and IL-17A. Each small *gray square* represents the production of one of the six cytokines as the layout indicates. **c** The increased frequencies of CD4+CD25+Foxp3+ Tregs are shown. Upregulation of Tregs is defined as at least twice the percentages of Tregs in the medium-only control

The HPV16-specific proliferative responses and frequencies of CD4+CD25+Foxp3+ regulatory T-cells (Tregs) after 7-day stimulation of PBMCs in both groups are shown in Fig. 1. Children in the case group were divided into five subgroups: (I) HPV DNA-positive placenta and detectable serum HPV antibodies during the first 2 months after birth; (II) HPV DNA-positive placenta without any HPV antibodies during the first 2 months; (III) HPV DNA-positive cord blood without an HPV DNA-positive placenta; (IV) persisting oral HPV (>24 months) during the active follow-up of 72 months, without an HPV DNA-positive placenta of cord blood; and (V) negative for oral HPV at all visits during the 72-month follow-up and testing oral HPV16-positive at the last visit when blood was taken for CMI. MRM-induced proliferation of the PBMCs was observed in all 19 case children, indicating that the PBMCs were capable of proliferating in response to the common recall antigens. HPV16-specific proliferative response was found in 14 of 19 case children, all showing a response to either both peptide pools of HPV16 E2 or the E2.2 pool only. Five of the case children were not responding to any of the HPV16 peptides. These non-responders include two children (ID305 and ID306), both with persistent oral HPV16 without any detectable HPV antibodies. The other two non-responders, ID101 and ID204, were found to be HPV-negative at the last time point, but were previously positive for HPV39 and HPV6, respectively. The fifth non-responder (ID011) had stayed negative for oral HPV at earlier visits during the 72-month follow-up, but was HPV6-seropositive during the first 2 months of life and tested HPV16-positive at the final visit. The percentage of the HPV16-specific Tregs was increased when compared to background frequencies (medium control) in two case groups children: ID101 had slightly elevated Treg frequency against the E6.4 peptide pool and ID303 against the E6.3 peptide pool.

In the control group, the PBMCs of 13 of 14 children responded by proliferation to MRM. The child without an MRM response (ID006), however, showed reactivity to the HPV16 peptides. Altogether, 12 of 14 control children displayed reactivity to E2, of whom eight had a proliferative response to both HPV16 E2 peptide pools. Five children responded to the E6 peptides and two to the E7.2 peptide pools. Two control children (ID001 and ID010) were negative in the proliferation test for HPV16. As controls, they did not show any oral HPV positivity during the follow-up, but were HPV-seropositive (HPV6 and 16 or HPV6, respectively). Increased percentages of Tregs specifically responding to HPV16 peptides were observed in four control children. In three children (ID002, ID008, and ID010), these Treg frequencies were relatively high and were induced by several peptide pools.

On the whole, the proliferative response induced to the HPV16 E6 and E7 oncoproteins was significantly different between the cases and controls, with a higher frequency in the control group than in the case groups (*p* = 0.047). The responses to the early antigen E2 were not significantly different between the groups. Similarly, no differences were found when the responses were stratified by HPV seropositivity (children with HPV antibodies detectable at 1 or 2 months and children with HPV antibodies after 2 months of age).

#### Cytokine polarization analysis

Supernatants of the proliferation tests were collected at day 6 and subjected to a cytometric bead array to analyze the cytokines produced. The selected cytokines IFN-γ, TNF-α, IL-2, IL-4, IL-5, IL-10, and IL-17A were measured to canvass the cytokine profile and to assess the presence of IL-17-producing HPV16-specific CD4+ T-cells. Generally, the levels of all cytokines measured were low (in most cases <100 pg/mL), but the range was wide (0.27–230.0 pg/mL, mean 6.3 pg/mL). The cytokine secretion was higher in the cultures displaying cell proliferation after stimulation than in the other cultures. IFN-γ, IL-10, and IL-2 were the most frequently detected cytokines in all children, followed by IL-17A, IL-5, and TNF-α. The concentrations of IFN-γ and IL-5 were slightly higher than that of other cytokines. Similar to our previous study, IL-4 was not detectable except in one case child (ID303) as a response to the E6.2 peptide pool.

**Fig. 2:**
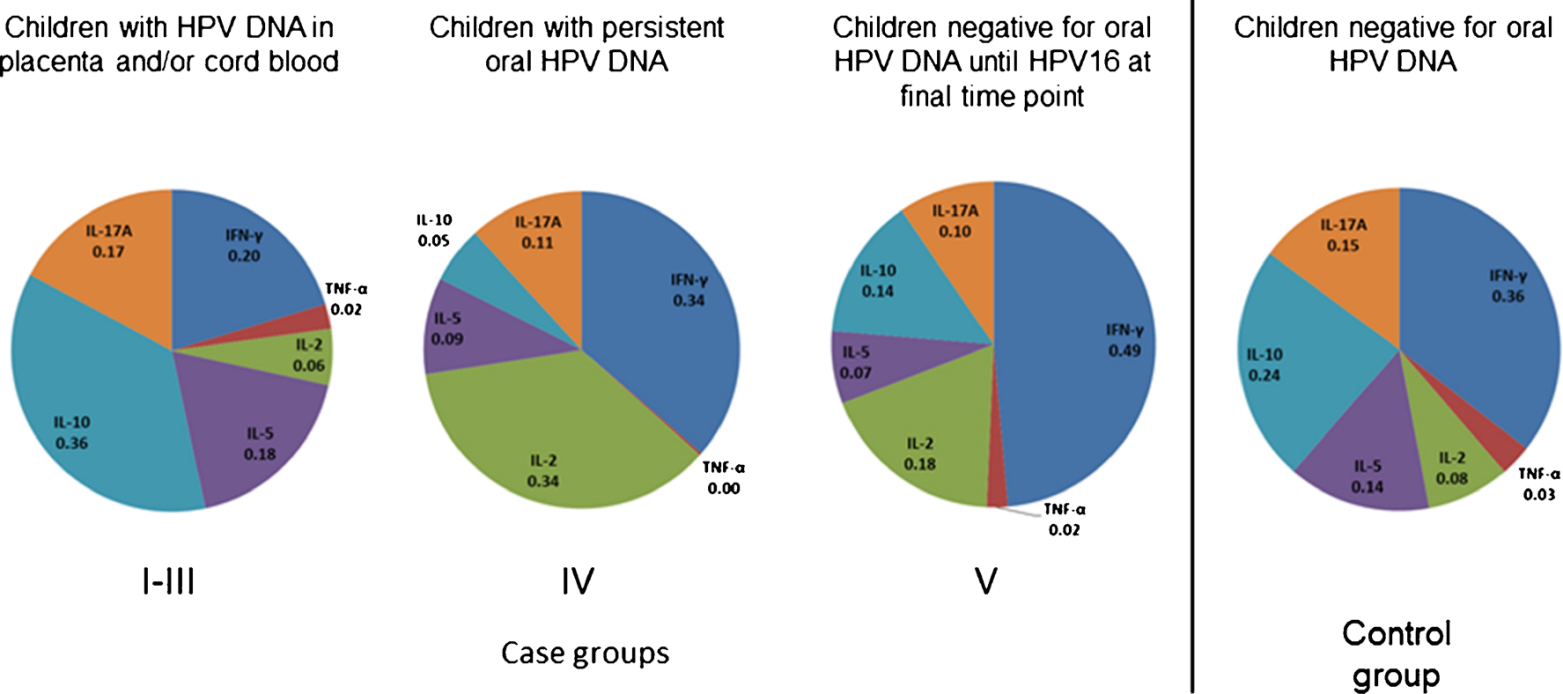
The mean fraction of each cytokine from the total amount of cytokine secretion among each subgroup. The fractions for each cytokine produced by each individual against all tested peptide pools were determined, and the mean of those fractions of whole subgroup were calculated

Similar to the proliferation data, the cytokine concentrations were compared between the five case subgroups and the control group. No differences between the groups were found when the cytokine secretion by the proliferative PBMC cultures was analyzed. However, when we considered the data of all peptide-stimulated PBMC cultures, there was a significantly higher HPV16 E2-induced production of IL-5 (*p* = 0.043), IL-10 (*p* = 0.013), and IL-17A (*p* = 0.043) in groups I–III (children with HPV DNA in placenta and/or cord blood) than in other case subgroups. Furthermore, when the children who tested HPV-positive in the placenta and/or cord blood (groups I–III) were stratified by their oral HPV persistence, those with no persistence showed higher TNF-α (*p* = 0.013), IL-2 (*p* = 0.009), and IL-5 (*p* = 0.006) secretion after E2.1 stimulation (pooled data). Figure 2 shows the mean concentrations of each cytokine and illustrates the distinct cytokine pattern in each group. The children born to HPV DNA-positive placenta and/or HPV-positive cord blood appear to show a shift towards cytokines IL-5 (type 2 cytokine) and IL-10 and IL-17A compared to other subgroups and the control group.

Overall evaluation of the results singled out two children who might be considered as having the characteristics of an HPV-tolerant individual. Child ID305 had had persistent oral HPV16 at two time points during the follow-up and tested HPV16-positive also at the last visit with HPV6 coinfection. Importantly, this child had no detectable HPV antibodies during the follow-up and, further, had no response to any of the HPV16 peptides. The mother of this child was negative for oral and genital HPV during the follow-up, including the final time point.

Child ID306 had had oral HPV16 detected at two time points (24 and 36 months) also without any HPV16 antibodies and no response against HPV16 peptides. No HPV DNA was detected in the oral sample at the final visit, which, however, does not entirely exclude the presence of oral HPV. The mother of this child had genital and oral HPV16 detected once (at 24 months) and persistent genital HPV45, but no HPV antibodies at any time point. Both children were born by vaginal delivery and the breast milk tested HPV DNA-negative after delivery. Clinical examination revealed normal oral mucosa at the 72 months visit.

## Discussion

In this study, we examined the HPV16 E2-, E6-, and E7-specific immune responses in children having distinct HPV profiles during the first 6 years of their lives. We found CMI responsiveness for E2 peptides in the majority of all tested children (78.8% of 33 children). This responsiveness was similar regardless of the child’s HPV status during the follow-up. The CMI responses against HPV16 E6 and E7 peptides were detected in 24.2% of children and were significantly more frequent in children who stayed constantly negative for oral HPV than in children with oral, placental, or cord blood HPV DNA. Interestingly, the analysis of the secretion patterns of Th1 and Th2 cytokines as a response to HPV16 peptides indicated more pronounced Th2 response in children born with HPV DNA-positive placenta and/or HPV-positive cord blood.

Our previous studies [13, 15] were the first to demonstrate that HPV16-specific immune responses exist among unvaccinated and sexually inexperienced children aged between 10 and 14 years. We found that 93.5% (29/31) of such children carried blood lymphocytes responsive to peptides overlapping the amino acid sequences of HPV16 E2 and E6 proteins. Unexpectedly, the HPV-related disease (CIN1–3) of the mother did not show any significant impact on the HPV16-specific immune responses of her child, when children born to HPV-negative mothers were used as the reference [15]. These results prompted us to further evaluate the possible effect of very early or long-term exposure to HPV on the appearance of HPV-specific CMI responses compared to children without HPV infection.

The cytokine polarization of HPV16 E2-, E6-, and E7-stimulated PBMCs was determined to canvass the possible alterations between types 1 and 2 cytokines. As the defects in the immune response against HPV have been linked with an increase in type 2 cytokines [3], it was reasoned that a comparison of the cytokine secretion patterns in children exposed to HPV in the early stages of their lives with HPV-negative children might reveal some differences. Since our previous observations implicating the important role of the placenta in the transmission of HPV [14, 25], the children with HPV DNA detected in placenta and/or cord blood were of special interest. Indeed, we found significantly higher HPV16 E2-induced levels of IL-5, IL-10, and IL-17A in placenta/cord blood HPV-positive children than in other case subgroups, indicating a more dominant Th2 effector or regulatory response, and further raising questions about the conceivable effects of HPV exposure during the prenatal period.

Earlier studies in mice have shown that inoculation of newborn mice with high doses of murine retrovirus induces type 2 cytokine responses, while low viral doses induce type 1 responses. Further, the initial viral dose with relation to the number of potentially responsive T-cells is critical for the induction of Th1 and Th2 responses and development of protective immunity in newborn mice. Thus, the lower T-cell number in neonates compared to adults explains the reduced immune responses to same antigen [6, 21, 26].

However, normal pregnancy in humans is known to be associated with Th2 skewing and the Th2 cytokine-dominated milieu at the placenta was suggested to establish a kind of protective homeostasis [27]. This Th2 predominance persists in the newborn’s system after birth for at least several months before the realignment of the Th1/Th2 environment [1, 5]. Thus, the continuation of this Th2 skewing or delaying of Th1 maturation is associated with atopy and other allergic diseases [1]. According to our results, one could hypothesize that HPV infection or exposure to viral antigens during the prenatal period might affect the activation and type of immune responses during later encounters with HPV and, further, even the outcome of the infection, which would, however, need a longer follow-up in order to be solved.

As in our previous observations, HPV16 E2 peptides were, once again, the most antigenic epitopes, inducing proliferative response similarly among the cases and the controls. The proliferative response to HPV16 E6 peptides was also found frequently. The responsiveness of control children to E2 and E6 peptides is in line with the previous observations in healthy adults [3, 4, 11, 38], while the proliferative responses of case children resemble more the pattern of mothers with CIN1–3 in our previous study [15]. Since HPV-specific immunity in children has not been reported in any other studies apart from our previous works, any comparisons of the current results are precluded.

Because HPV E2 is the main factor in the transcription initiation and regulation of viral genes and, therefore, abundant in all HPV-infected cells at the stage of viral amplification, the individual’s responsiveness to HPV16 E2 peptides alludes to the existence of circulating HPV16 E2-specific memory T-cells, originating from prior or present replicative HPV16 infection [17]. Furthermore, a cross-reactivity of HPV16 E2, E6, and E7 peptides with T-cells specific to other closely related HPV types is conceivable, albeit a rarely observed event [3, 4, 33, 40].

Interestingly, we could identify two case children (ID305 and ID306) with persistent oral HPV16 (HPV DNA detected in repeated samples over 24 months) who showed no response to any HPV16 peptides. In general, the measured cytokine production was below the cut-off values as expected, since no HPV16-specific T-cell proliferation was detected in these children. Both of them had demonstrated oral HPV16 persistence during their active follow-up without HPV16 antibodies, and ID305 had oral HPV16 DNA detected also at the last (15 years) visit. This child had also received three doses of bivalent HPV vaccination, while child ID306 was not vaccinated. At the moment, we cannot ascertain whether the repeatedly detected DNA of a given HPV type in the oral mucosa is a sign of persistent HPV infection or if it is a new encounter with the same HPV type. However, our recent (unpublished) data on persistent oral HPV16 infections in adults have shown that HPV16 is either in an integrated or mixed physical state, confirming the concept of HPV persistence. In the same study, all subjects who cleared their oral infection had HPV16 in an episomal state (Lorenzo et al., unpublished). It is tempting to consider that children who received prophylactic HPV vaccination and who show persistent oral HPV16 infection might have an integrated HPV16, undetectable by the antibodies elicited by the HPV vaccination. In addition, it might well be that these two children, especially ID305, carrying persistent oral HPV16 without any detectable immunological responses, can have a specific defect in their immune system or even conceivable features of HPV tolerance. We also noticed that, in children with persistent HPV during the follow-up, the same HPV type was detected already at birth or during the first 2 months of life, implying that these very early infections could be more prone to remaining persistent. In addition, another child (ID011) in the case group showed no HPV16-specific response, but still had HPV16 DNA detectable in the oral mucosa. In this case, one possible explanation for the lack of immune response could be a very recent infection, not yet recognized by the adaptive immune system [29]. This particular child had not been vaccinated against HPV before CMI testing. The remaining four children without HPV16-specific CMI had no oral HPV16 detected during the follow-up.

To conclude, these results support our earlier observations on the existence of HPV16-specific CMI in children [13, 15]. It seems that the vast majority of children carry CD4+ memory T-cells responsive to peptides of HPV16. Accordingly, these observations suggest that all these children had been exposed to HPV16 during their early lives, regardless of their oral HPV status at each time point during follow-up. However, a substantial proportion of children who remained oral HPV-negative during follow-up showed also response against the HPV16 E6 protein, suggesting an importance of HPV16 E2- and E6-induced CMI in the successful control of HPV infection. In turn, the unresponsiveness to HPV16 peptides seen in some children with persistent oral HPV16 might suggest a kind of specific defect in their immune system. The more dominant Th2 cytokine profile in cases who were infected early during life insinuates that either the viral dose or the specific Th2 microenvironment installed by the placenta may have a significant role in steering the type of HPV-specific immunity in young children.

Altogether, the present findings of HPV16-specific CMI, together with our earlier data on the natural history of HPV infections during infancy and childhood, attest the view that an early exposure to HPV might be highly relevant for the subsequent HPV infections in adulthood, and also have some influence on the effectiveness of prophylactic HPV vaccines.
